# Supplementary material for: Potentially Toxic Planktic and Benthic Cyanobacteria in Slovenian Freshwater Bodies: Detection by Quantitative PCR
Source: Toxins (Basel). 2021 Feb 11;13(2):133. doi: 10.3390/toxins13020133 (PMC7917684; doi:10.3390/toxins13020133)
Supplement: Supplementary file 1 [file toxins-13-00133-s001.zip › Supplementary file S6.pdf]

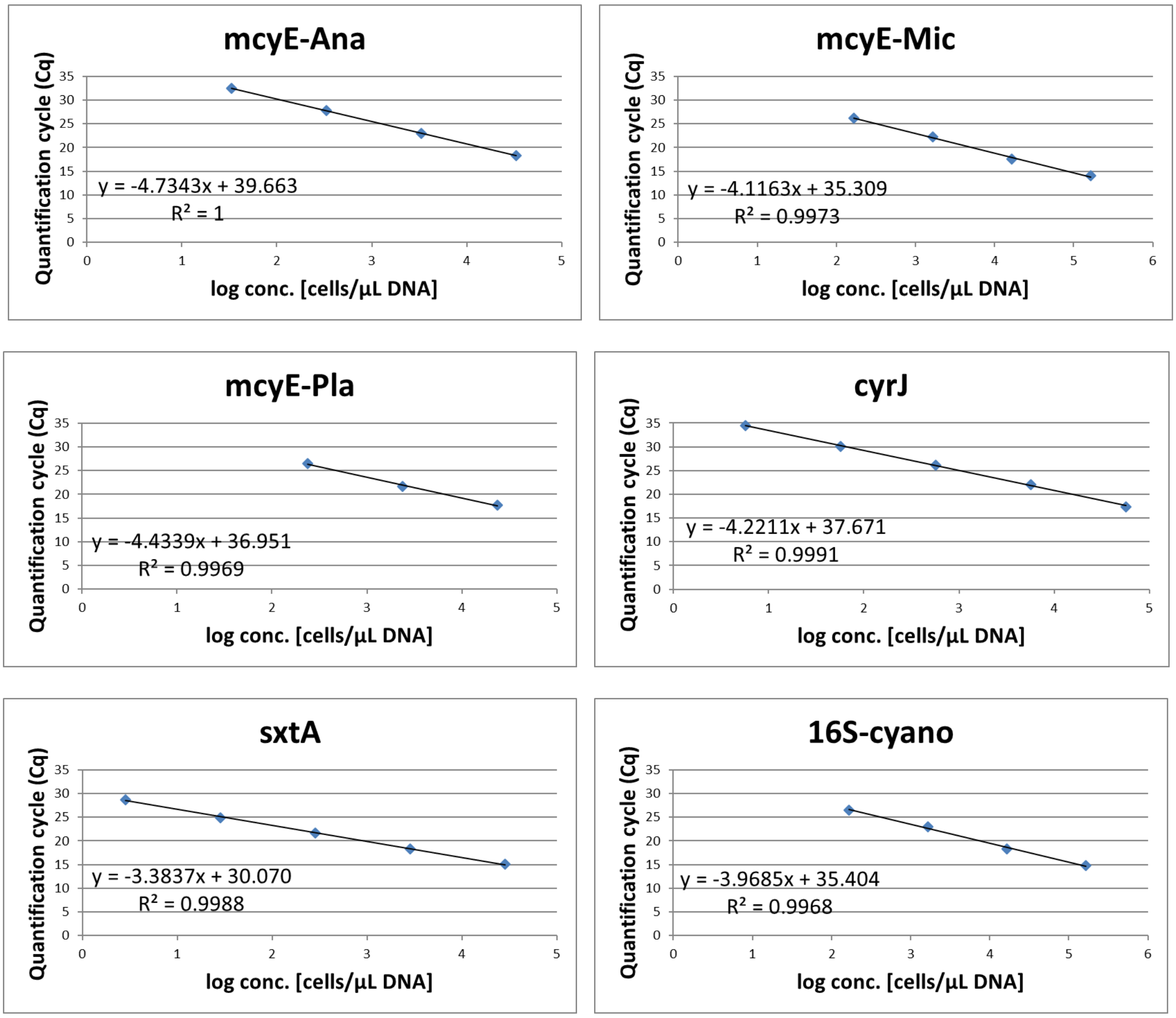

Supplementary figure S6: Linear ranges of calibration curves for selected qPCR assays based on dilutions of reference cyanobacterial cultures DNA.
